# LASSBio-897 Reduces Lung Injury Induced by Silica Particles in Mice: Potential Interaction with the A_2A_ Receptor

**DOI:** 10.3389/fphar.2017.00778

**Published:** 2017-10-31

**Authors:** Vinicius F. Carvalho, Tatiana P. T. Ferreira, Ana C. S. de Arantes, François Noël, Roberta Tesch, Carlos M. R. Sant’Anna, Eliezer J. L. Barreiro, Carlos A. M. Fraga, Patrícia M. Rodrigues e Silva, Marco A. Martins

**Affiliations:** ^1^Laboratório de Inflamação, Instituto Oswaldo Cruz, Fundação Oswaldo Cruz, Rio de Janeiro, Brazil; ^2^Laboratório de Farmacologia Bioquímica e Molecular, Instituto de Ciências Biomédicas, Universidade Federal do Rio de Janeiro, Rio de Janeiro, Brazil; ^3^Programa de Pós-Graduação em Farmacologia e Química Medicinal, Instituto de Ciências Biomédicas, Universidade Federal do Rio de Janeiro, Rio de Janeiro, Brazil; ^4^Laboratório de Avaliação e Síntese de Substâncias Bioativas, Instituto de Ciências Biomédicas, Universidade Federal do Rio de Janeiro, Rio de Janeiro, Brazil; ^5^Departamento de Química, Instituto de Ciências Exatas, Universidade Federal Rural do Rio de Janeiro, Rio de Janeiro, Brazil

**Keywords:** A_2A_ receptor, cAMP, fibrosis, LASSBio-897, silicosis

## Abstract

Silicosis is a lethal fibro-granulomatous pulmonary disease highly prevalent in developing countries, for which no proper therapy is available. Among a small series of *N*-acylhydrazones, the safrole-derived compound LASSBio-897 (3-thienylidene-3, 4-methylenedioxybenzoylhydrazide) raised interest due to its ability to bind to the adenosine A_2A_ receptor. Here, we evaluated the anti-inflammatory and anti-fibrotic potential of LASSBio-897, exploring translation to a mouse model of silicosis and the A_2A_ receptor as a site of action. Pulmonary mechanics, inflammatory, and fibrotic changes were assessed 28 days after intranasal instillation of silica particles in Swiss–Webster mice. Glosensor cAMP HEK293G cells, CHO cells stably expressing human adenosine receptors and ligand binding assay were used to evaluate the pharmacological properties of LASSBio-897 *in vitro*. Molecular docking studies of LASSBio-897 were performed using the genetic algorithm software GOLD 5.2. We found that the interventional treatment with the A_2A_ receptor agonist CGS 21680 reversed silica particle-induced airway hyper-reactivity as revealed by increased responses of airway resistance and lung elastance following aerosolized methacholine. LASSBio-897 (2 and 5 mg/kg, oral) similarly reversed pivotal lung pathological features of silicosis in this model, reducing levels of airway resistance and lung elastance, granuloma formation and collagen deposition. In competition assays, LASSBio-897 decreased the binding of the selective A_2A_ receptor agonist [^3^H]-CGS21680 (IC_50_ = 9.3 μM). LASSBio-897 (50 μM) induced modest cAMP production in HEK293G cells, but it clearly synergized the cAMP production by adenosine in a mechanism sensitive to the A_2A_ antagonist SCH 58261. This synergism was also seen in CHO cells expressing the A_2A_, but not those expressing A_2B_, A_1_ or A_3_ receptors. Based on the evidence that LASSBio-897 binds to A_2A_ receptor, molecular docking studies were performed using the A_2A_ receptor crystal structure and revealed possible binding modes of LASSBio-897 at the orthosteric and allosteric sites. These findings highlight LASSBio-897 as a lead compound in drug development for silicosis, emphasizing the role of the A_2A_ receptor as its putative site of action.

## Introduction

Silicosis is one of the most important occupational diseases in both developed and developing countries, and is characterized by an irreversible inflammatory process into the lungs caused by inhalation of free crystalline silica (Leung et al., 2012). Although the prevalence of silicosis is decreasing in some developed countries (Bang et al., 2015), this disease is still very common in developing nations (Nelson et al., 2010), particularly in mining countries (Rees and Murray, 2007; Tse et al., 2007). In Brazil, a prevalence of 20 new annual cases of silicosis per 100,000 exposed people was reported (Ministry of Health, 2010). Silicosis pathogenesis is described as a complex interaction between different cell types and inflammatory mediators, leading to nodular lesions formation, fibrosis and reduction of lung elasticity (Ferreira et al., 2013; Franklin et al., 2016). After phagocytosis of silica particles, macrophages orchestrate this complex response through the release of inflammatory and fibrogenic cytokines. These mediators stimulate granuloma formation and lung fibroblasts to produce extracellular matrix components, culminating to the development of fibrosis (Fazzi et al., 2014). Once fibrosis has developed, there is no effective treatment for silicosis. Thus, the search for new compounds becomes indispensable for the management of this disease.

**FIGURE 1:**
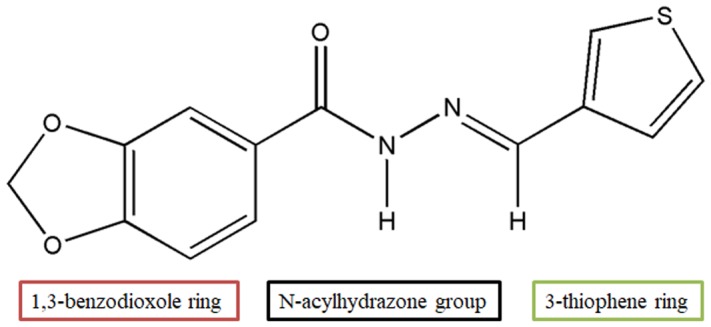
Molecular structure of 3,4-methylenedioxybenzoyl-3-thienylhydrazone (LASSBio-897).

3,4-Methylenedioxybenzoyl-3-thienylhydrazone (LASSBio-897) (**Figure 1**) is a new *N*-acylhydrazone bioactive derivative synthesized from safrole, a Brazilian natural product that could be obtained in high yield from sassafras oil. LASSBio-897 was designed as a regiomeric analog of 3,4-methylenedioxybenzoyl-2-thienylhydrazone (LASSBio-294) (Gonzalez-Serratos et al., 2001), through a substitution of 2-thienyl ring to the bioisosteric 3-thienyl ring (Zapata-Sudo et al., 2010). LASSBio-294 induces vasodilatation in rat aortic rings (Silva et al., 2002), reduces blood pressure in spontaneously hypertensive rats (SHR), improves diastolic activity in myocardial infarction rats and inhibits collagen deposition and heart fibrosis (Costa et al., 2010). LASSBio-897 was about 200-fold more potent than LASSBio-294 causing vasodilation of rat aortic rings and significantly reduced blood pressure values in SHR (Zapata-Sudo et al., 2010). While trying to assess the spectrum of pharmacological activity in multiple targets, we submitted 10 μM LASSBio-897 to a screening of targets in the Cerep’s “Diversity Profile” platform (Poitiers, France). Under this condition, it displayed an inhibitory activity ≥ 30% at only 3 of the 98 potential targets investigated, which were A_2A_ receptor (72%), 5-HT transporter (56%), and NE transporter (42%).

The A_2A_ receptor is an adenosine receptor that is coupled to Gs-protein and its activation results in increased levels of intracellular cAMP (Antonioli et al., 2015). Adenosine is a purine nucleoside involved with preservation and restoration of tissue homeostasis, in several sites, including lung (Chen et al., 2013). Adenosine receptors, such as A_2A_ receptors, are widely distributed in various organs, including lungs, as well as fibroblasts and immune cells (Factor et al., 2007; Koupenova and Ravid, 2013; Eisenstein and Ravid, 2014). Activation of A_2A_ receptors induces a strong anti-inflammatory response by reduction of adherence, chemotaxis and phagocytosis of neutrophils, and reduction of pro-inflammatory cytokines generation and oxidative stress (Lappas et al., 2005). The activation of A_2A_ receptors inhibits inflammatory response in different models of lung diseases, including murine models of asthma, lipopolysaccharide-driven airway neutrophilic infiltration and cigarette smoke-induced chronic obstructive pulmonary disease (Fozard et al., 2002; Bonneau et al., 2006). In the current study, we evaluated the effect of LASSBio-897 on silica-induced lung inflammation and fibrosis. We also investigated the interaction of this bioactive compound with A_2A_ receptors.

## Materials and Methods

### Synthesis of 3,4-Methylenedioxybenzoyl-3-Thienylhydrazone (LASSBio-897)

LASSBio-897 was synthesized at Laboratório de Avaliação de Substâncias Bioativas of Federal University of Rio de Janeiro, Brazil. The methodology used in the synthesis of LASSBio-897 was previously reported by our research group (Zapata-Sudo et al., 2010) and was reproduced in this work.

### Silicosis Induction and Treatment

Male Swiss mice weighing 18–20 g were obtained from the breeding colonies of Oswaldo Cruz Foundation and used in accordance with the guidelines of the Committee on Use of Laboratory Animals of the Oswaldo Cruz Foundation (CEUA-FIOCRUZ, license L-034/09). Mice were housed in groups of five at 25^o^C on a 12 h day/night cycle and fed with a standard sterile diet of mouse chow and water was allowed *ad libitum*. Silicosis was induced by intranasal instillation with 10 mg crystalline silica particles (SiO_2_; Sigma Chemical Co, St. Louis, MO, United States; particle size 0.5–10 μm) under halothane volatile anesthesia (Cristália, São Paulo, Brazil). A control group received sterile saline (0.9% NaCl) intranasally. All analyses were performed 28 days post-silica instillation. LASSBio-897 was given by gavage and CGS 21680 was given intraperitoneally, once a day for 7 days, starting at the 21st day post-silica particle provocation. Non-treated silicotic mice received an equal volume of vehicle (0.1% DMSO, oral route). All *in vivo* analyses were repeated twice with the exception of the one involving CGS 21680 treatment.

### Invasive Assessment of Respiratory Mechanics

Following general anesthesia with nembutal^®^ (60 mg/kg, i.p.), tracheostomy and neuromuscular blockade (pancuronium bromide, 1 mg/kg, i.v.), the mechanic ventilator was connected to the mouse through an endotracheal tube. Then, transpulmonary resistance and elastance were assessed 28 days after silica particle intranasal instillation by using invasive whole-body plethysmography (Buxco Electronics, United States). Mice were allowed to stabilize for 5 min and increasing concentrations of methacholine (3, 9, and 27 mg/ml) were aerosolized for 5 min each. Baseline pulmonary mechanics parameters were assessed with aerosolized phosphate-buffered saline (PBS) (Ferreira et al., 2013).

### Lung Histology

Left lungs from each experimental group were dissected and placed in Millonig fixative solution (pH = 7.4) with 4% paraformaldehyde for 48 h to preserve tissue architecture. Tissues were dehydrated and clarified in xylene before paraffin embedding. Lung sections of 4 μm were stained with hematoxylin and eosin (H&E), to analyze granulomas, or Picrosirius, to evaluate collagen deposition, in light microscope using 3DHISTECH–Pannoramic MIDI whole slide scanner (capture with a 20× objective lens) and the resulting images analyzed with CaseViewer 3.3, Pannoramic Viewer 1.15.4, and HistoQuant softwares (3DHISTECH). Silica crystals were analyzed, in 15 independent fields, with a light microscope (Olympus BX51) equipped with polarizing attachment for detecting birefringent particles and the quantification were performed using the software Image-Pro Plus Version 6.2 (Media Cybernetics Inc., Rockville, MD, United States).

### Lung Cytokine and Chemokine Quantification

Murine IL-6, TNF-α, and macrophage inflammatory protein-2 (MIP-2/CXCL-2) levels were measured in right lung samples by means of ELISA kit (R&D Systems, Minneapolis, MN, United States) as previously described (Ferreira et al., 2016). The results were expressed as picograms of cytokine per right lung.

### Collagen Evaluation

For quantification of collagen, the right lung was homogenized in 0.05 M Tris + 1 M NaCl (pH = 7.4), containing protease inhibitor (Hoffmann-La Roche Ltd., Switzerland). Total soluble collagen was extracted overnight at room temperature and centrifuged for 1 h at 15,000 ×*g*. The supernatant was used to evaluate collagen levels by means of Sircol^TM^ kit (Biocolor Ltd., Newtownabbey, United Kingdom) following manufacturer’s guidelines. The results were expressed as mg collagen per right lung.

### Immunohistochemistry Staining

Paraffin-embedded sections of lungs were deparaffinized, rehydrated, and boiled in 10 mM urea for 15 min to enhance antigen retrieval. Tissue sections were incubated with 3% H_2_O_2_ for 10 min to block endogenous peroxidase. To prevent non-specific binding, the sections were then incubated for 2 h with a solution containing 5% bovine serum albumin (BSA) dissolved in Tris-buffered saline enriched with 0.1% Tween 20 (TBST). Sections were incubated overnight at 4°C with the specific antibody (monoclonal mouse anti-mouse α-SMA or monoclonal rat anti-mouse F4/80 from Sigma–Aldrich, St. Louis, MO, United States) and Serotec, Kidlington, United Kingdom, respectively) diluted in TBST with 1% BSA. Primary antibody binding was detected after incubating the sections with a horseradish peroxidase conjugated-secondary antibody (polyclonal anti-mouse IgG HRP, R&D System, Minneapolis, MN, United States) and polyclonal anti-rat IgG HRP, Serotec, Kidlington, United Kingdom) for 2 h at 4°C, followed by a 15 min exposure to enzyme substrate 3-amino-9-ethylcarbazole (AEC). Sections were washed with TBST between all steps and weakly counterstained with hematoxylin for easy identification of tissue structures. In negative controls, primary antibody was omitted and tissues were incubated with antibody diluent only. The images were captured through light microscope (Olympus BX51) coupled to a video camera (Olympus DP72), and analyzed with software Image Pro Plus 6.2 (Media Cybernetics Inc., Rockville, MD, United States). The number of positive pixels was divided by the field area and expressed as pixels/μm^2^.

### Binding Assays

The capacity of LASSBio-897 to bind to A_2A_ receptors was assessed using classical competition assays at equilibrium, in two different conditions. In the first assay, we used 150 μg protein of a rat striatum membrane preparation, rich in A_2A_ receptors (Cunha et al., 1996), and the agonist [^3^H]-CGS21680 (10 nM) as radioligand, in experimental conditions reported by Luthin and Linden (1995), i.e., incubation during 2 h at 4°C in a medium containing MgCl_2_ 5 mM, EDTA-Na_2_ 1 mM, and Tris-HCl 50 mM(pH 7.4). For the second assay, we used 10 μg protein of a membrane preparation of HEK293 cells overexpressing the human A_2A_ receptors (RBHA2AM400UA, PerkinElmer, United States). In this assay, we used the endogenous agonist [^3^H]-adenosine (25 nM) as the radioligand. The incubation was performed at 25°C for 1 h in a medium containing EDTA-Na_2_ 1 mM, MgCl_2_ 10 mM, an adenosine deaminase inhibitor [*erythro*-9-(2-Hydroxy-3-nonyl) adenine hydrochloride-EHNA, 10 μM], an adenosine uptake inhibitor (nitrobenzylthioinosine-NBI, 3 μM) and Tris-HCl 50 mM (pH 7.4). For both assays, the non-specific binding was estimated using 30 μM NECA and the filters were rapidly washed with cold Tris-HCl 5 mM (pH 7.4), either 3 × 4 mL ([^3^H]-CGS21680) or 2 × 2 mL ([^3^H]-adenosine). For the assay with [^3^H]-adenosine, the glass fiber filters (GMF 3, Filtrak, Germany) were previously soaked in 0.5% polyethyleneimine. For exploring the effect of LASSBio-897 on [^3^H]-ZM241385 dissociation kinetics, rat striatal membranes were incubated at 25°C in a medium containing [^3^H]-ZM241385 0.5 nM, EDTA-Na_2_ 1 mM and Tris-HCl 50 mM (pH 7.4). After 45 min, the dissociation of the [^3^H]-ZM241385-receptor complex was initiated by addition of NECA 30 μM with or without 30 μM LASSBio-897.

### Cell Culture

We used Glosensor cAMP Human Embryonic Kidney 293 (HEK293G) cells (Promega, Madison, WI, United States), which is a cell line that stably expresses the biosensor variant encoded by the pGloSensor^TM^-20F cAMP plasmid; and Chinese Hamster Ovary cells stably expressing both the human A_1_ (CHO-A1), A_2A_ (CHO-A2A), A_2B_ (CHO-A2B), or A_3_ (CHO-A3) receptor and a reporter gene, Secreted Placental Alkaline Phosphatase (SPAP), under the transcriptional control of a six CRE promoter (McDonnell et al., 1998). Cells were used until the 30th passage. HEK293G were maintained in Dulbecco’s modified Eagle’s medium (DMEM) while all CHO cell lines were maintained in DMEM/Nutrient mix F12 (DEMEM/F12). All culture mediums were supplemented with 10% fetal calf serum (FCS) and 2 mM L-glutamine and all cell lines were grown at 37°C in a humidified 5% CO_2_: 95% air atmosphere.

### GloSensor cAMP Assay

The GloSensor^TM^ cAMP assay is an extremely sensitive technique to detect changes in intracellular concentration of cAMP using a live-cell system in a non-lytic format. The assay uses genetically encoded biosensor variants with cAMP binding domains fused to mutant forms of *Photinus pyralis* luciferase, that promote large increases in light output. The analysis of kinetic measurements of cAMP in HEK293G cells was performed following the manufacturer’s instructions (Promega, Madison, WI, United States). Briefly, cells were grown to 90% confluence in 96-well plates. Then, the media was removed and replaced with 100 μL of 6% v/v dilution of GloSensor^TM^ cAMP reagent stock solution in HBSS, and the cells incubated for 2 h. LASSBio-897 and/or agonists (50 μL each, diluted in HBSS) were then added to each well concomitantly, and the plate was then read at 37°C in an EnVision Luminometer plate reader (PerkinElmer, Akron, OH, United States) in a kinetic mode, from 10 s to 1 h. The peak light emission in the time course of the reaction was recorded. In the experiments where we used antagonists or inhibitors, they were added 30 min before stimulation.

### CRE-Mediated SPAP Transcription Measurement

CRE-dependent SPAP reporter activity was evaluated as previously described (Baker et al., 2010). Briefly, CHO-A1, CHO-A2A, CHO-A2B, or CHO-A3 cells were incubated with LASSBio-897 and/or adenosine for 6 h at 37°C in a 5% CO_2_: 95% atmosphere air. In the case of CHO-A1 and CHO-A3 cells, we added forskolin (3 μM) 30 min after stimulation with adenosine. CRE-dependent SPAP gene transcription was quantified by color change caused by hydrolysis of *p*-nitrophenol phosphate, using MRX plate reader (Dynatech Labs, Chantilly, VA, United States) at 405 nm absorbance.

### Molecular Docking

The crystal structure of the A_2A_ receptor with PDB code 4EIY (Liu et al., 2012) was chosen to perform molecular docking studies of LASSBio-897, using the genetic algorithm software GOLD 5.2 (CCDC). The number of genetic operations (crossover, migration, mutation) in each docking run used in the searching procedure was set to 100,000. Docking analyzes were performed on the orthosteric site and on the allosteric site. The binding sites were selected to include all amino acid residues located within a 10 Å distance from the cocrystalized ligand (ZM241385) at the orthosteric site and a 10 Å distance from the sodium ion at the allosteric site. The docking runs at the orthosteric site were done allowing complete flexibility only for the ligand. However, even in the rigid protein mode, the program optimizes hydrogen bond geometries by rotating hydroxyl and amino groups of amino acid side chains. At the allosteric site, some amino acid side chains were allowed to stay flexible during the docking runs. This was done because the LASSBio-897 molecule occupies a higher volume than the sodium ion, thus the flexibility of amino acid side chains could enable a better fit of the molecule into this site. The resulting poses were classified according to the *GoldScore* fitness score function (Jones et al., 1997).

### Statistical Analysis

Results were expressed as mean ± SD. All data were analyzed in blind flashing and evaluated to ensure normal distribution. Statistical analysis was performed with one-way ANOVA followed by the multiple comparison test of Newman–Keuls–Student. In lung mechanical experiments, statistical analysis was performed with two-way ANOVA followed by the multiple comparison test of Bonferroni. The *P*-values ≤ 0.05 were considered statistically significant. In binding experiments at equilibrium, we used the model of competition for one binding site (top and bottom fixed at 100% and 0%, respectively) for analysis of the specific binding data by non-linear regression analysis (GraphPad Prism 5) and estimation of the IC_50_ (concentration of the unlabeled ligand that inhibits the binding of the radioligand by 50%).

## Results

### CGS 21680 Inhibits Silica-Particle Induced AHR

**FIGURE 2:**
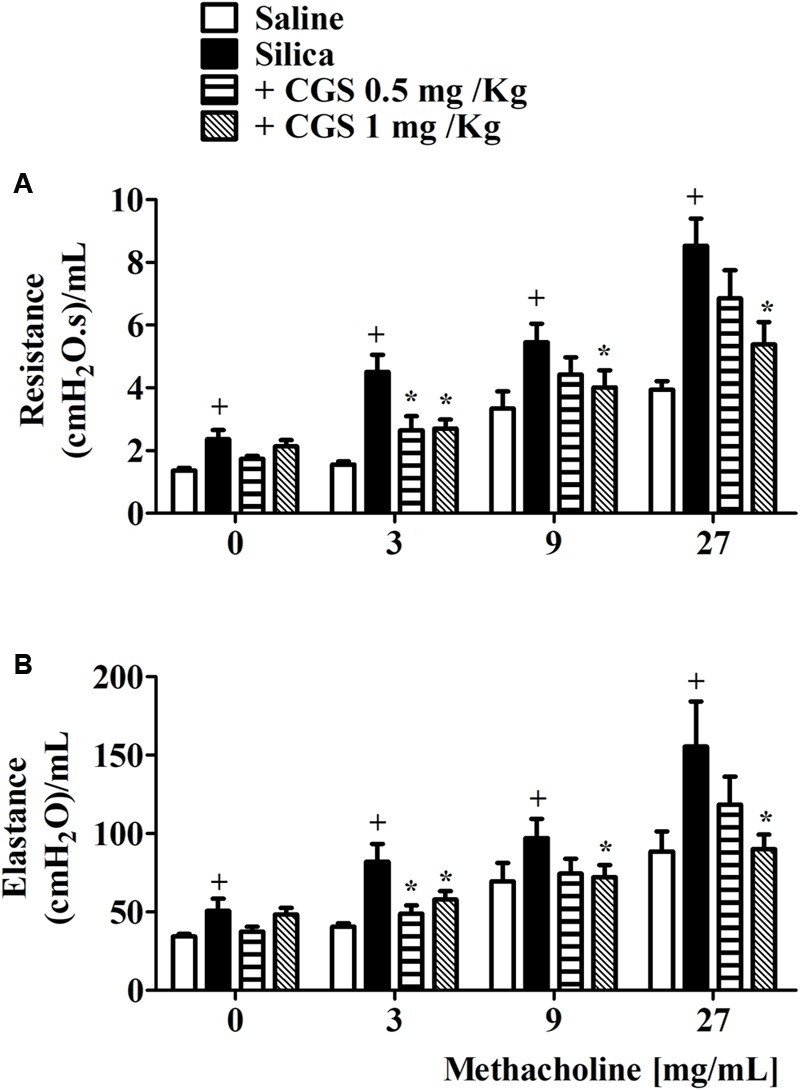
CGS 21680 inhibits airway hyper-reactivity (AHR) in silicotic mice. Silica (10 mg) was given i.n. and analyses were performed 28 days later. Intraperitoneal treatment with LASSBio-897 was performed daily for 7 days, starting 21 days after silica instillation. AHR was induced by provocation with increasing concentrations of aerosolized methacholine and measured as airway resistance **(A)** and lung elastance **(B)** parameters. Silicotic non-treated animals received an equal amount of vehicle (DMSO 0.1%). Data are expressed as the means ± SD from at least seven mice. ^+^*P* < 0.05 compared to saline-provoked group. ^∗^*P* < 0.05 compared to silica-provoked group. CGS = CGS 21680.

While investigating whether or not the A_2A_ receptor would be a relevant therapeutic target in silicosis, we studied the effect of the interventional intraperitoneal treatment with the selective A_2A_ agonist CGS 21680 (0.5 or 1 mg/kg) on AHR triggered by silica particle intranasal instillation in mice. As illustrated in **Figure 2**, values of airway resistance and lung elastance following aerosolized methacholine (3–27 mg/mL) were elevated 28 days after silica instillation, compared to negative control mice challenged with saline (silica particle vehicle), pointing out a state of airway hyper-reactivity (AHR) in line with previous studies (Tripathi and Pandey, 2010). CGS 21680 treatment completely reversed silica-induced AHR regarding both airway resistance (**Figure 2A**) and lung elastance changes (**Figure 2B**) at dose of 1 mg/kg, being partially effective at 0.5 mg/kg.

### LASSBio-897 Effects on Lung Function, Granuloma Formation, and Collagen Deposition in the Lung Parenchyma

**FIGURE 3:**
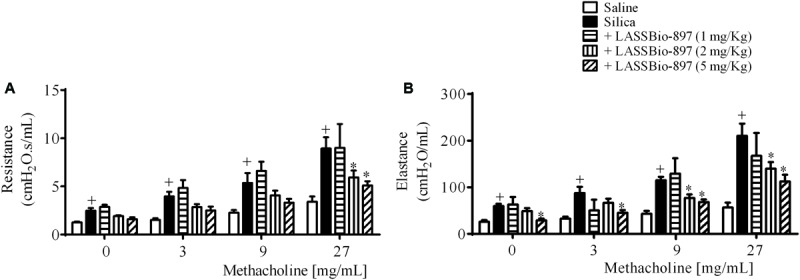
LASSBio-897 inhibits airway hyper-reactivity (AHR) in silicotic mice. Silica (10 mg) was given i.n. and analyses were performed 28 days later. Oral treatment with LASSBio-897 was performed daily for 7 days, starting 21 days after silica instillation. AHR was induced by provocation with increasing concentrations of methacholine and measured as airway resistance **(A)** and lung elastance **(B)** parameters. Silicotic non-treated animals received an equal amount of vehicle (DMSO 0.1%). Data are expressed as the means ± SD from six mice. These results are representative of two independent assays. ^+^*P* < 0.05 compared to saline-provoked group. ^∗^*P* < 0.05 compared to silica-provoked group.

In order to evaluate the effect of the interventional treatment with LASSBio-897 on pathological changes triggered by silica particle intranasal instillation in mice, we gave the compound orally (1–5 mg/kg) once a day for 7 days. As in the case of the CGS 21680 treatment, the treatment started at day 21 post-silica, when lung inflammatory changes and other dysfunctions were already established as reported (Ferreira et al., 2013; Trentin et al., 2015). As shown in **Figure 3**, LASSBio-897 reversed silica-induced AHR regarding both airway resistance (**Figure 3A**) and lung elastance changes (**Figure 3B**) at doses of 2 and 5 mg/kg given orally, being inactive at 1 mg/kg.

**FIGURE 4:**
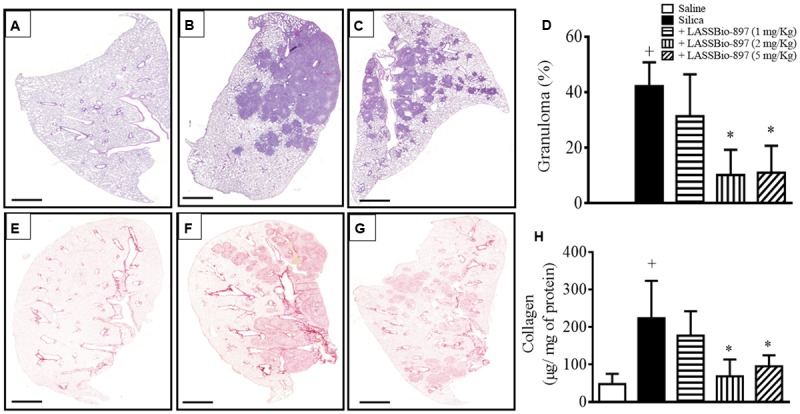
LASSBio-897 decreases lung inflammation and fibrosis in silicotic mice. Silica (10 mg) was given i.n. and analyses were performed 28 days later. Oral treatment with LASSBio-897 was performed daily for 7 days, starting 21 days after silica instillation. Histological sections of mouse lungs provoked with silica **(B,F)** and treated with LASSBio-897 (5 mg/kg) **(C,G)**. Animals instilled with PBS were used as controls **(A,E)**. **(D)** Morphometric analyses of granuloma. **(H)** Quantitative analysis of collagen content in the lung tissue was performed by Sircol technique. Silicotic non-treated animals received an equal amount of vehicle (DMSO 0.1%). Data are expressed as the means ± SD from six mice. Scale bars = 200 μm. These results are representative of two independent assays. ^+^*P* < 0.05 compared to saline-provoked group. ^∗^*P* < 0.05 compared to silica-provoked group.

Histological evaluation of lung tissue samples, 28 days after intranasal instillation of silica particles, showed a parenchymal accumulation of inflammatory cells associated with granuloma formation (**Figure 4B**) and increased collagen deposition (**Figure 4F**), findings which were not observed in mice subjected to intranasal instillation of saline (**Figures 4A,E**, respectively). LASSBio-897 treatment of silica-stimulated mice, at oral doses of 5 mg/kg, resulted in a significant reduction in granulomatous and fibrogenic responses, as illustrated by the representative histological sections, shown in **Figures 4C,G**, respectively. Quantitative assessments revealed that silica-induced granuloma (**Figure 4D**) and collagen deposition (**Figure 4H**) were equally inhibited by LASSBio-897 at doses of 2 mg/kg and 5 mg/kg, but they were not modified at the dose of 1 mg/kg.

### LASSBio-897 Effects on the Number of Macrophages, Myofibroblasts, and Silica Particles Present in the Lung Parenchyma

**FIGURE 5:**
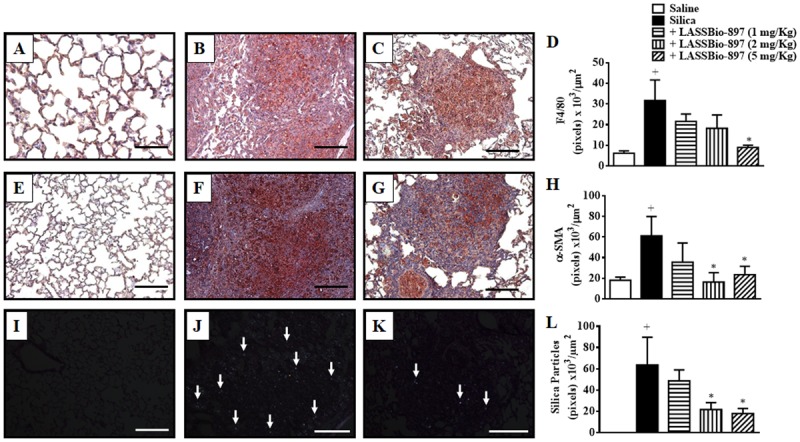
LASSBio-897 reduces macrophages, myofibroblasts, and silica particles into the lungs of silicotic mice. Silica (10 mg) was given i.n. and analyses were performed 28 days later. Oral treatment with LASSBio-897 was performed daily for seven consecutive days, starting 21 days after induction of silicosis. Panels show representative photomicrographs of lung sections of F4/80 immunoreactivity (upper panels), α-SMA immunoreactivity (middle panels), and silica particles (lower panels) of saline-provoked mice **(A,E,I)**; silica-provoked mice **(B,F,J)**; silica-provoked mice treated with LASSBio-897 (5 mg/kg) **(C,G,K)**. **(D)** and **(H)** represent the quantification of pixels associated with F4/80 and α-SMA expression, respectively. **(L)** Quantitative analysis of silica particles. Silicotic non-treated animals received an equal amount of vehicle (DMSO 0.1%). White arrows indicate silica particles in **(J)** and **(K)**. Data are expressed as the means ± SD from six mice. Scale bars = 200 μm. These results are representative of two independent assays. ^+^*P* < 0.05 compared to saline-provoked group. ^∗^*P* < 0.05 compared to silica-provoked group.

We assessed the effect of LASSBio-897 on the number of macrophages and myofibroblasts in the lung parenchyma of mice exposed to silica, as attested by the immunohistochemistry of lung sections for F4/80 and α-SMA, respectively. Silicotic mice showed an increase in the levels of F4/80^+^ cells (**Figure 5B**) and myofibroblasts (**Figure 5F**) accumulated in granuloma areas, compared to samples from negative control mice (**Figures 5A,E**, respectively). Oral treatment of silicotic mice with LASSBio-897 (5 mg/kg) reduced the immune reactivity for F4/80 (**Figure 5C**) and α-SMA (**Figure 5G**), suggesting a substantial inhibitory effect upon the levels of infiltrating macrophages and myofibroblasts. Quantitative data for the impact of LASSBio-897 (1–5 mg/kg) upon F4/80 (**Figure 5D**) and α-SMA (**Figure 5H**) pointed out that immune reactivity for α-SMA were inhibited at the doses of 2 and 5 mg/kg, while F4/80 expression were reduced only at 5 mg/kg, and both changes remaining unaltered at 1 mg/kg.

Furthermore, under light microscopy, in a system equipped with polarizing filters, we visualized silica crystals present in the lung parenchyma sections from silicotic mice (**Figure 5J**). Histological sections from mice stimulated with saline were employed as negative controls (**Figure 5I**). Remarkably, LASSBio-897 (5 mg/kg) notably reduced the amount of silica crystals identified in the lung tissue (**Figure 5K**). **Figure 5L** shows that LASSBio-897, at 2 and 5 mg/kg but not at 1 mg/kg, significantly inhibited the amount of silica dispersed in the lung.

### LASSBio-897 Effects on the Lung Tissue Production of Pro-Inflammatory Mediators

We also examined the effect of LASSBio-897 on lung tissue production of key inflammatory mediators in silicosis. We noted that mice reacted to silica challenge with increased lung tissue levels of TNF-α, IL-6, and MIP-2/CXCL-2, as compared with samples from control mice. The interventional treatment with LASSBio-897 (2 and 5 mg/kg) significantly inhibited the silica-induced augmentation in the levels of the assessed pro-inflammatory cytokines (**Table 1**).

**Table 1:** Effect of LASSBio-897 on cytokine/chemokine generation in the lung tissue of silicotic mice.

Cytokine (pg/lung tissue)	Saline	Silica	Silica + LASSBio-897 (2 mg/kg)	Silica + LASSBio-897 (5 mg/kg)
TNF-α	77 ± 25	486 ± 81^+^	175 ± 90^∗^	171 ± 47^∗^
MIP-2	763 ± 171	16119 ± 6553^+^	3000 ± 1050^∗^	4258 ± 1189^∗^
IL-6	123 ± 20	415 ± 199^+^	161 ± 76^∗^	223 ± 96^∗^

*LASSBio-897 (2 and 5 mg/mouse) was given 21 days after silica instillation (10 mg) and the analyses were performed 24 h post-silica. Values represent the mean ± SD. These results are representative of two independent assays. ^+^*P* < 0.05 vs. saline-challenged group; ^∗^*P* < 0.05 vs. silica-challenged group.*

### LASSBio-897 Is a Ligand of A_2A_ Receptors

**FIGURE 6:**
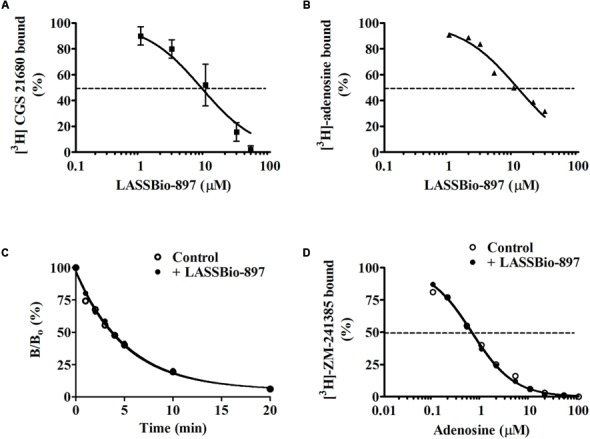
LASSBio-897 binds at the orthosteric site of A_2A_ receptors, but has no negative allosteric effect. LASSBio-897 inhibits [^3^H]-CGS21680 binding in rat striatal membranes **(A)** and [^3^H]-adenosine binding in a membrane preparation of human A_2A_ receptors **(B)** in a concentration-dependent manner. The specific binding of the radioligands are expressed as percent of the control without competing drug. The fitted curves were obtained by non-linear regression analysis using the model of competition for one binding site, as detailed in the methods. **(C)** Effect of LASSBio-897 (30 μM) on [^3^H]-ZM241385 dissociation kinetics in rat striatal membranes. After 45 min incubation at 25°C, the dissociation of the [^3^H]-ZM241385-receptor complex was initiated by addition of NECA 30 μM with or without 30 μM LASSBio-897. The fitted curves were obtained by non-linear regression using the model of one phase exponential decay. In this experiment, the specific binding of the control corresponds to 95% of the total binding. **(D)** Effect of LASSBio-897 (10 μM) on the competition curve of adenosine for [^3^H]-ZM-241385 binding in rat striatal membranes. The incubation was performed at 25°C during 2 h. Specific binding of [^3^H]-ZM-241385 is expressed as percent of binding in the absence (control) or presence of 10 μM LASSBio-897. At this concentration, LASSBio-897 inhibits the binding of [^3^H]-ZM-241385 by only 13% and the specific binding corresponds to 88% of the total binding. Data are from two **(A)**, or one **(B–D)**, experiments performed in triplicate.

Initially, we tested the effect of LASSBio-897 in a classical competition assay using rat striatum membranes. In these conditions, saturation experiments with [^3^H]-CGS21680 revealed a single class of specific binding sites with a *B*_max_ of 1070 ± 90 *f*mol/mg protein and a *K*_d_ of 207 ± 55 nM. **Figure 6A** shows that LASSBio-897 decreased the specific binding of [^3^H]-CGS21680 in a concentration-dependent manner, with an IC_50_ of 9.3 μM (95% confidence interval (CI): 6.4–15.3 μM). In the experiments reported here, the total, non-specific and specific CPM (counts per min) of the control were 374, 213, and 161, respectively (mean of two experiments). As differences exist in binding affinity and potency of some agonists between human and rat (Kull et al., 1999), we decided to test the effect of LASSBio-897 on the binding of the endogenous agonist [^3^H]-adenosine to human A_2A_ receptors. As shown in **Figure 6B**, a similar result was obtained with LASSBio-897 decreasing the binding of the agonist radioligand with an IC_50_ of 11.4 μM (95% CI: 9.0–14.4 μM). In the experiment performed with membranes of cells transfected with the human receptor, the total, non-specific and specific CPM of the control were 990, 151, and 799, respectively. To verify if these effects of LASSBio-897 were due to competition at the orthosteric site and not to a negative allosteric modulation (alternative hypothesis for such results), we used two different approaches using [^3^H]-ZM-241385 as radioligand and the rat striatal membrane preparation, for economical and practical reasons. Indeed, this classical selective radioligand for the A_2A_ receptor allows a much better signal than [^3^H]-CGS21680 or [^3^H]-adenosine in this preparation (in our conditions, the specific binding corresponds to around 90% of the total binding) and has been used successfully in different protocols in our laboratory (Noel and do Monte, 2017). In the first experiment, we used the most classical, and more sensitive, approach for investigating directly an allosteric effect, the radioligand dissociation assay (Hill et al., 2014), already used by us in previous studies (Lopes et al., 2004). **Figure 6C** shows that LASSBio-897 did not increase the dissociation kinetics of the orthosteric ligand (here [^3^H]-ZM-241385) as expected for a negative allosteric modulator. The second assay is more indirect but allows addressing a putative allosteric effect that could be detected only with the endogenous orthosteric ligand (probe dependency). **Figure 6D** shows that 10 μM LASSBio-897 did not modify the affinity of adenosine for its binding to the A_2A_ receptor, measured indirectly from a competition assay with the antagonist [^3^H]-ZM-241385.

### LASSBio-897 Is an Activator of A_2A_ Receptors

**FIGURE 7:**
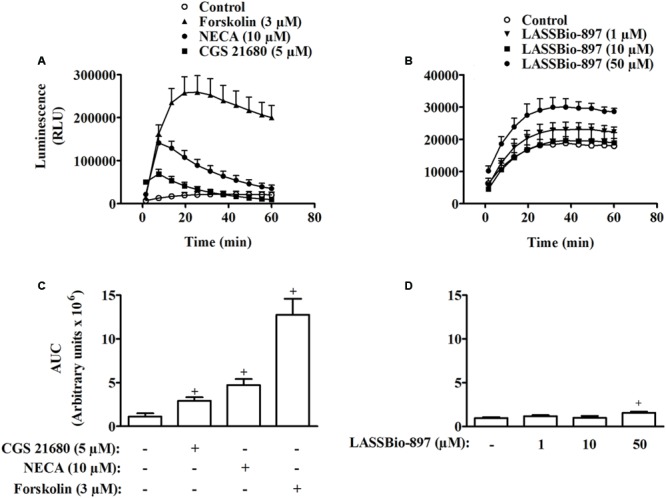
LASSBio-897 acts as a weak partial agonist of A_2A_ receptors. **(A)** and **(C)** shows HEK293G cells stimulated with 3 μM forskolin, 5 μM CGS 21680 or 10 μM NECA. **(B)** and **(D)** shows different concentrations of LASSBio-897. Immediately after stimulation, the kinetic traces for cAMP generation were then measured by a luminometer. AUC (10 s–60 min) were calculated from the analyses of cAMP production induced by forskolin, CGS 21680, or NECA **(C)** and LASSBio-897 **(D)**. Control group received an equal amount of vehicle (DMSO 0.1%). Data points are means ± SD of triplicate determinations from a single experiment and are representative of three experiments. ^+^*P* < 0.05 compared to non-stimulated cells.

**FIGURE 8:**
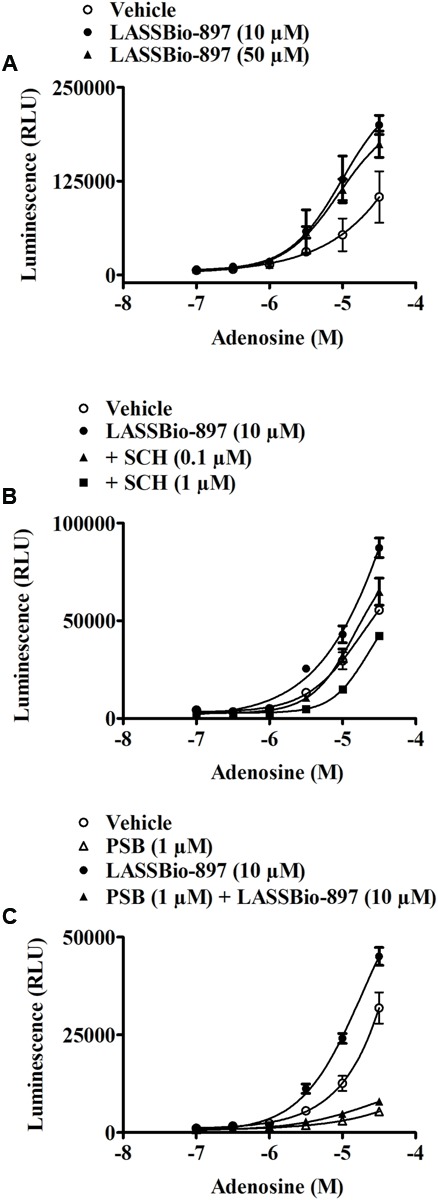
LASSBio-897 enhances activation of A_2A_ receptors evoked by adenosine in HEK293G cells. **(A)** HEK293G cells were concomitantly stimulated with LASSBio-897 and adenosine. HEK293G cells were treated with an A_2A_ receptor antagonist **(B)**, or A_2B_ receptor antagonist **(C)** 30 min before the simultaneous addition of LASSBio-897 and adenosine. Immediately after stimulation, the kinetic traces for cAMP generation were measured by a luminometer. Control group received an equal amount of vehicle (DMSO 0.1%). Data points are means ± SD of triplicate determinations from a single experiment and are representative of three experiments. SCH, SCH 58261. PSB, PSB 603.

In HEK293G cells, stimulation with 3 μM forskolin, 10 μM NECA, or 5 μM CGS21680 induced a fast production of high amounts of intracellular cAMP (**Figures 7A,C**). In contrast, LASSBio-897 produced a small increase of intracellular cAMP levels, and only at the highest concentration used (50 μM) (**Figures 7B,D**). However, the co-incubation with LASSBio-897 (10 and 50 μM) increased the cAMP production induced by 10 and 30 μM adenosine (**Figure 8A**). Treatment with the A_2A_ receptor selective antagonist SCH 58261 blocked the potentiating effect of LASSBio-897 on adenosine-induced cAMP production in a concentration dependent manner (**Figure 8B**). However, although treatment with A_2B_ receptor selective antagonist PSB 603 drastically decreases adenosine-induced cAMP production, addition of LASSBio-897 slightly increases the cAMP concentration, suggesting that its potentiating effect is not mediated by the A_2B_ receptor (**Figure 8C**).

### LASSBio-897 Does Not Alter Adenosine Metabolism, But Acts Selectively on Adenosine A_2A_ Receptor

**FIGURE 9:**
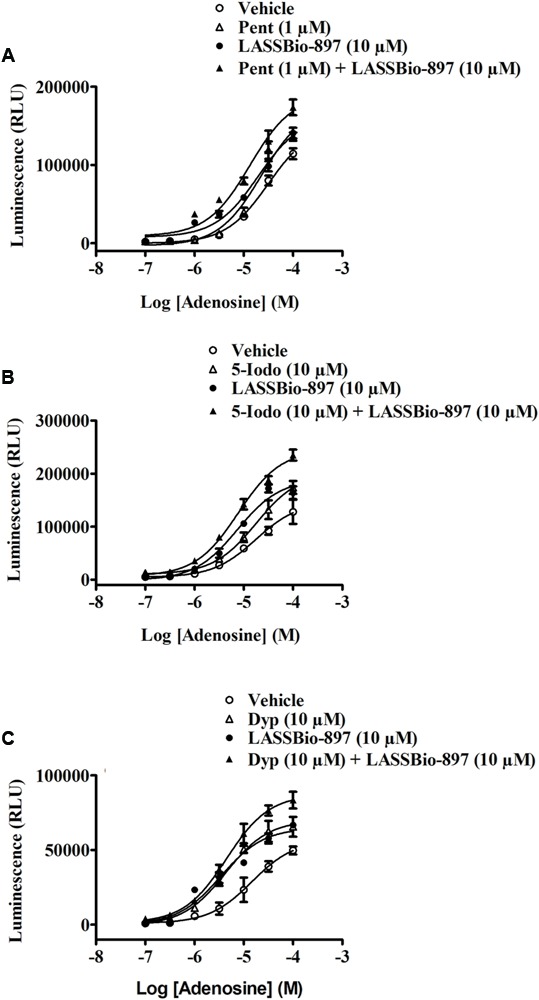
Adenosine enhancer activity of LASSBio-897 does not depend of adenosine metabolism inhibition in HEK293G cells. HEK293G cells were treated with adenosine deaminase inhibitor **(A)**, adenosine kinase inhibitor **(B)**, or adenosine reuptake blocker **(C)** 30 min before simultaneous addition of LASSBio-897 and adenosine. Immediately after stimulation, the kinetic traces for cAMP generation were measured by a luminometer. Control group received an equal amount of vehicle (DMSO 0.1%). Data points are means ± SD of triplicate determinations from a single experiment and are representative of three experiments. Pent, pentostatin; 5-Iodo, 5-iodotubercidin; Dyp, dipyridamole.

In order to assess whether LASSBio-897 potentiating effect upon cAMP production by adenosine could be due to inhibition of either adenosine metabolism or transport, we pre-incubated HEK293G cells for 30 min with inhibitors of two enzymes, which metabolize adenosine, as well as with an inhibitor of adenosine reuptake transporters. Individually, both LASSBio-897 and adenosine deaminase inhibitor pentostatin increased the effect of 100 μM adenosine on intracellular cAMP production (**Figure 9A**). When used together, pentostatin and LASSBio-897 produced a greater increase of the effect of 100 μM adenosine (**Figure 9A**). Similarly, both LASSBio-897 and adenosine kinase blocker 5-iodotubercidin increased the effect of 100 μM adenosine on cAMP production. The association of 5-iodotubercidin with LASSBio-897 produced an even greater increase of the effect of 100 μM adenosine (**Figure 9B**). Finally, we noted that the inhibitor of adenosine transporters dipyridamole also increased the effect of 100 μM adenosine similarly to LASSBio-897. Once more, co-incubation with both dipyridamole and LASSBio-897 produced a greater increase of the effect of adenosine (**Figure 9C**).

**FIGURE 10:**
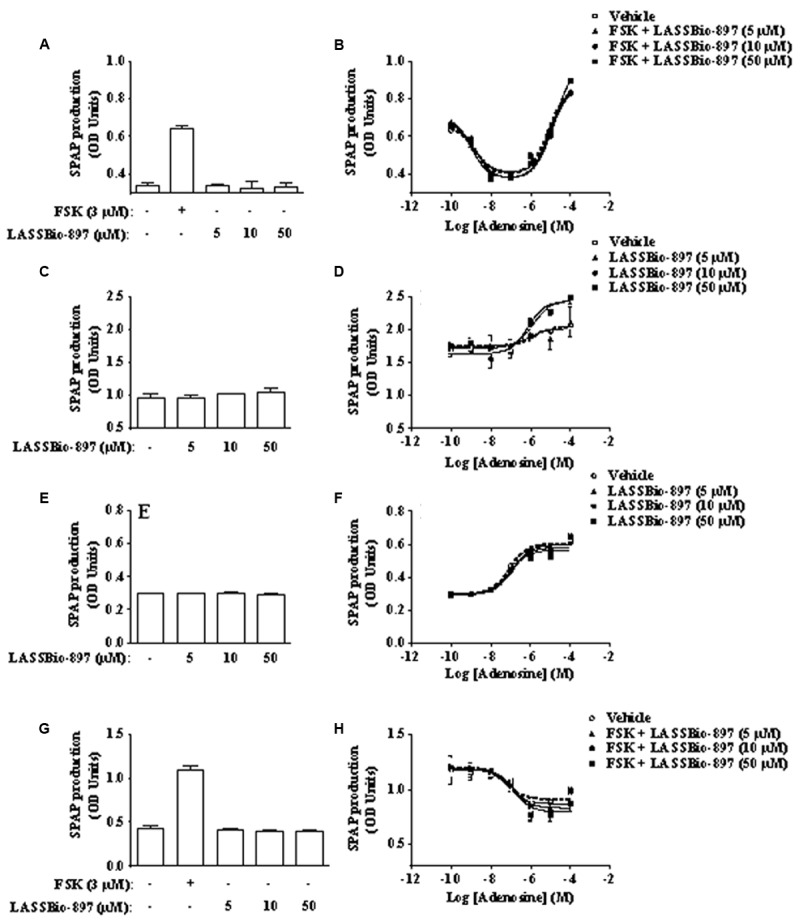
LASSBio-897 enhances the stimulatory effect of adenosine on cAMP production in A_2A_, but not A_1_, A_2B_, and A_3_ receptors CHO-transfected cells. The experiments were performed with CHO cells transfected with reporter gene secreted SPAP and human A_1_ **(A,B)**, A_2A_ **(C,D)**, A_2B_ **(E,F)**, or A_3_ **(G,H)** receptors. Cells were concomitantly stimulated with different concentrations of LASSBio-897 and adenosine. Six hours after stimulation, CRE-mediated SPAP transcription was measured by spectrophotometry. The bars represent SPAP secretion from non-stimulated cells. Control group received an equal amount of vehicle (DMSO 0.1%). Data points are means ± SD of triplicate determinations from a single experiment and are representative of three experiments. FSK, forskolin.

In order to address the selectivity of LASSBio-897 effect for the four subtypes of adenosine receptors, we used an assay based on CRE-mediated gene transcription (Baker et al., 2010). To observe the activation of A_1_ and A_3_ receptors, we added forskolin in order to raise the basal adenylyl cyclase activity and subsequent CRE-gene transcription activation as shown in **Figures 10A,G**, respectively. In CHO-A1 transfected cells, we observed that adenosine induced a biphasic response in the presence of forskolin. First, there was an inhibitory Gi-mediated suppression of CRE gene transcription, while at higher agonist concentration, a Gs-mediated enhancement of the response to forskolin was noted (**Figure 10B**). LASSBio-897 did not alter the effect of adenosine on A_1_ (**Figure 10B**), A_2B_ (**Figures 10E,F**), and A_3_ receptors (**Figure 10H**) at any concentration analyzed. In CHO-A2A transfected cells, LASSBio-897 enhanced the *E*_max_ of adenosine for its effect on CRE gene transcription (**Figures 10C,D**).

### LASSBio-897 Can Bind to the Orthosteric and Allosteric Sites of A_2A_ Receptor

**FIGURE 11:**
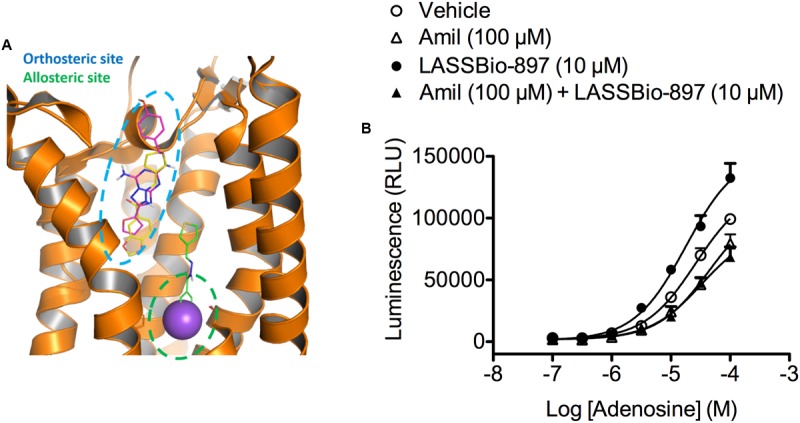
Evidence for participation of the Na^+^ allosteric site in the effect of LASSBio-897 at the A_2A_ receptor. **(A)** Overlapping of best docking solutions of LASSBio-897 showing the interaction region at the orthosteric site (carbon atoms in yellow) and at the allosteric site (carbon atoms in green). The ZM241385 ligand (carbon atoms in magenta) and sodium ion (purple) are shown for comparison. **(B)** HEK293G cells were treated with amiloride 30 min before stimulation simultaneously with LASSBio-897 and adenosine. Immediately after stimulation, the kinetic traces for cAMP generation were then measured by a luminometer. Control group received an equal amount of vehicle (DMSO 0.1%). Data points are mean ± SD of triplicate determinations from a single experiment and are representative of three experiments. Amil, amiloride.

The molecular docking results showed that LASSBio-897 is predicted to be able to interact favorably both at the orthosteric and allosteric sites. **Figure 11A** indicates that LASSBio-897 could replace the co-crystallized ligand ZM-241385 at the orthosteric site without approaching the sodium ion pocket. The main interaction observed in the orthosteric site solution involved Asn253. As expected from its greater volume, the solutions at the allosteric pocket indicate that LASSBio-897 occupies the sodium ion binding site and also part of the orthosteric site (**Figure 11A**). The main interaction observed at this site was with Ser91, a residue directly implied in the interaction with the sodium ion at the allosteric pocket.

In order to test the hypothesis of LASSBio-897 binding to the allosteric site of A_2A_ receptor, we treated HEK293G cells with amiloride, a negative allosteric modulator of adenosine receptors. Amiloride alone reduced the effect of 100 μM adenosine, and blocked the potentiating effect of LASSBio-897 on adenosine-induced cAMP production (**Figure 11B**).

## Discussion

Prior studies have highlighted the anti-inflammatory properties of some compounds of the class of *N*-acylhydrazones, but the mechanism of action remains poorly understood (Fraga and Barreiro, 2006; Kummerle et al., 2012). The present work investigated the therapeutic potential of the *N*-acylhydrazone LASSBio-897 in experimental silicosis and explored its putative mechanism of action.

Our results revealed that the interventional treatment with LASSBio-897 or the A_2A_ receptor agonist CGS 21680 reversed silica-induced AHR in mice. LASSBio-897 also inhibited the progress of major pathological changes triggered by nasal-instilled silica particles, including granuloma formation and collagen deposition, in the mouse lung parenchyma. The effect appeared associated with a reduction in the content of F4/80^+^macrophages, α-SMA^+^ myofibroblasts and crystalline silica particles in the lungs, in parallel with a significant decrease in the lung tissue levels of pro-inflammatory cytokines and chemokines. Binding studies suggested that LASSBio-897 has some affinity for the A_2A_ receptor and functional assays indicated that it is a weak activator of cAMP production in HEK293G cells. Also, LASSBio-897 increased the adenosine-induced cAMP production in cells expressing A_2A_ receptor, being inactive in cells transfected with A_1_R, A_2B_R, or A_3_R, through a mechanism unrelated to blockade of adenosine metabolism. Finally, docking studies indicated that LASSBio-897 could bind at both orthosteric and allosteric sites of A_2A_ receptor, which is in line with the ability of amiloride – a negative allosteric modulator of adenosine receptor – to prevent the synergistic effect of LASSBio-897 and adenosine. These findings suggest that LASSBio-897 is indeed a promising therapeutic strategy to control silicosis. It is likely to act at the A_2A_ receptor through a non-canonical mechanism based on the fact that native A_2A_ receptors are probably predominantly present as homodimers (Ferre et al., 2014). Although merely putative, we hypothesize that LASSBio-897 could act as a bitopic ligand (Lane et al., 2014) where binding at the allosteric site of one protomer (considering a dimer composition), would increase the efficacy of adenosine at the other protomer (cAMP assays). On the other hand, binding of LASSBio-897 at the orthosteric site would explain the results of the binding competition assays (decrease of radiolabeled orthosteric ligand binding).

Alveolar macrophages display a pivotal role in the recognition, uptake, and clearance of environmental particulate matters that traffic into the airways (Blank et al., 2007). The pathogenesis of the silicosis is largely attributed to the direct damage by inhaled crystalized silica particles to alternatively activated macrophages localized in the airway wall (Kawasaki, 2015). When this barrier is broken, free silica crystals translocate into the interstitial space and are taken up by M1 macrophages, which eventually sequester the particulate matter in the lung (Huaux, 2007). By releasing pro-inflammatory cytokines and chemokines, the inflammatory macrophages are also involved in inducing granuloma formation, which further overlaps with an extensive fibrotic response throughout the lung interstitial area (Kawasaki, 2015). The characteristic features of human silicosis can be modeled in mice by intranasal instillation of crystalized silica particles (Ferreira et al., 2013, 2016; Trentin et al., 2015). LASSBio-897 (2 and 5 mg/kg), given orally from days 21–27 post-silica, significantly inhibited an extensive area of fibro-granulomatous formation, which reach about 40% of the lung parenchyma in non-treated mice 28 days post-challenge.

Because fibro-granulomatous lesions can take important part of the lung of patients with silicosis, a respiratory deficit is expected to occur in such patients. Actually, in mild silicosis, and particularly in the early phase of the disease, spirometry results are frequently negative. However, in more severe cases, increased airway resistance and residual volume have been reported in clinical studies (de Mesquita Junior et al., 2006; Leung et al., 2012; Sa et al., 2013). Using invasive whole-body plethysmography in mice exposed to silica particles, we noted that the LASSBio-897 treatment significantly reduced the elevated levels of lung resistance and elastance, also inhibiting the state of airway hyper-reactivity to the bronchoconstrictor methacholine as compared to non-treated silicotic controls. Similarly to the granuloma and fibrotic responses, also the bronchial hyper-reactivity noted in animal models of silicosis has been associated in a causative manner to inflammatory mediators generated locally (Tripathi and Pandey, 2010; Ferreira et al., 2013).

In attempt to further clarify the mechanism that underpins the protective effect of LASSBio-897 in this model, we found first that the treatment reduced the levels of inflammatory agents such as TNF-α, IL-6, and MIP-2/CXCL-2. These mediators are largely implicated in the recruitment and activation of macrophages and fibroblasts, which play a central role in the genesis of silica particle-induced tissue injury in the respiratory tract (Driscoll, 2000; Tripathi and Pandey, 2010; Fazzi et al., 2014). This is relevant since the granulomatous inflammation induced by silica particles consists predominantly of macrophages (Hamilton et al., 2008). In addition, myofibroblasts are responsible for the increased collagen production in silica-induced fibrosis (Huaux, 2007). In fact, we noted a significant decrease in the levels of F4/80 and α-SMA immunoreactive cells in lung tissue samples in those mice treated with the active dose of LASSBio-897 (5 mg/kg), which pointed out the decrease in the number of macrophages and myofibroblasts, respectively. Therefore, LASSBio-897 may exert its protective effect by interfering with both inflammatory and fibrotic responses. A further and important aspect of the effect on experimental silicosis is the decrease in the amount of crystals of silica dispersed in the lung interstitial space. It is noteworthy that silica particles can be drained through the lymphatic system into the lymph nodes, particularly when they are free enough to be carried out from the interstitial space (Ferreira et al., 2013). Thus, by reducing the silica-induced fibro-granulomatous response, LASSBio-897 may favor silica particle mobility and elimination from the lung parenchyma via lymphatic draining.

The screening of 10 μM LASSBio-897 at the 98 potential targets of the Cerep’s “Diversity Profile” platform (Poitiers, France), showed an inhibitory activity superior to 30% at only the A_2A_ receptor (72%), 5-HT transporter (56%), and NE transporter (42%). As activation of A_2A_ receptor is a potent inductor of anti-inflammatory response in several models of lung diseases (Bonneau et al., 2006), we utilized several *in vitro* and *in silico* models for investigating how LASSBio-897 could modulate A_2A_ receptor signaling. First, we showed that LASSBio-897 inhibited the binding of both [^3^H]-CGS21680, a selective A_2A_ receptor agonist, and [^3^H]-adenosine, the endogenous agonist, in rat striatum membranes and human A_2A_ receptors, respectively, indicating that LASSBio-897 either competes with the orthosteric ligand of the A_2A_ receptor or exerts a negative allosteric effect by decreasing the affinity of the radioligand. Note that these competition binding assays cannot estimate a putative effect of LASSBio-897 on the efficacy of the orthosteric ligands. The two experimental approaches used for addressing this question were able to discard the second hypothesis.

Then, we tested the ability of LASSBio-897 to modulate cAMP production *in vitro*. For this purpose, we choose the HEK293G cells since they express the A_2A_ receptors whose activation increases the intracellular levels of cAMP (Ongini et al., 1999). Only at the highest concentration (50 μM), LASSBio-897 induced an increase in cAMP production that was much smaller than that observed with NECA and CGS21680, suggesting that LASSBio-897 could be a weak partial agonist of the A_2A_ receptor. Surprisingly, LASSBio-897 increased the production of cAMP induced by adenosine, when the two drugs were co-incubated. In fact, if LASSBio-897 were only a classical partial agonist, it should decrease, and not increase, the effect of adenosine. In order to test if the activation of A_2A_ could be involved in the curative effect of LASSBio-897 in silica particle-induced lung injury, we evaluate the effect of selective A_2A_ agonist CGS 21680. CGS 21680 decreases silica particle-induced AHR, evidenced by the parameters of airway resistance and lung elastance, in a similar way to that seen with LASSBio-897 treatment, suggesting that the curative effect of LASSBio-897 on silica particle-induced lung injury can be related to the its ability in increase the effect of adenosine on A_2A_.

To challenge our hypothesis of a synergistic interaction between LASSBio-897 and adenosine at the A_2A_ receptor level, we explored three distinct approaches. Initially, we pre-treated HEK293G cells with the A_2A_ antagonist SCH 58261. This A_2A_ antagonist in both concentrations (0.1 and 1 μM) blocked the synergic effect of LASSBio-897 on adenosine-induced cAMP, suggesting that this compound in fact acts on A_2A_ receptor. Although we noted that SCH 58261 inhibited the synergic effect of LASSBio-897 in the range of low nanomolar concentrations, we evaluated the effect of LASSBio-897 on the A_2B_ receptor, once HEK293 cells also express the A_2B_ receptors (Cooper et al., 1997). We noted that LASSBio-897 maintained its ability to increase the production of cAMP induced by adenosine even in the presence of the A_2B_ antagonist PSB 603, suggesting that LASSBio-897 did not act on this receptor. Then, we pre-treated HEK293G cells with adenosine metabolism or transport blockers. We showed that inhibitors of adenosine deaminase (pentostatin), adenosine kinase (5-iodotubercidin) or adenosine transporters (dipyridamole) increased the synergistic effect of LASSBio-897 on adenosine-induced cAMP, indicating that our compound did not act blocking metabolism or reuptake of adenosine. Together, these data suggest that the effect of LASSBio-897 would not be due to increase of the supply of adenosine to the A_2A_ receptors. Next, we analyzed the selectivity of LASSBio-897 toward the A_2A_ receptors, since HEK293 cells express other receptors able to activate adenylyl cyclase, including the A_2B_ receptors (Cooper et al., 1997). To address this point, we used CHO cells transfected with A_1_, A_2A_, A_2B_, or A_3_ receptors. We observed that LASSBio-897 increased the adenosine-induced activation of A_2A_ but not of A_1_, A_2B_, and A_3_ receptors, indicating that LASSBio-897 selectively modulates the A_2A_ receptors.

**FIGURE 12:**
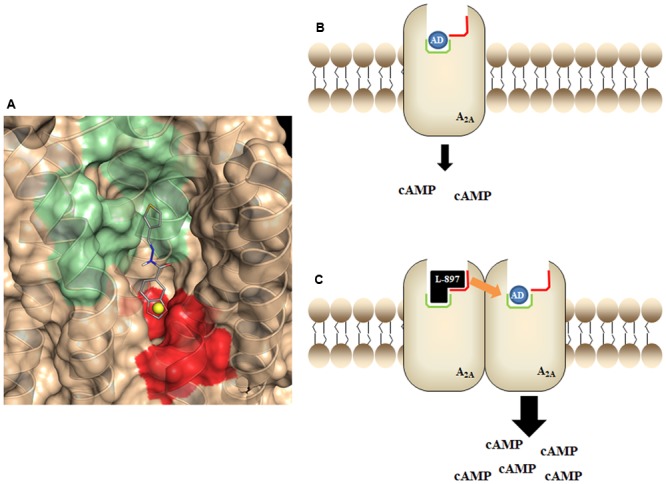
Schematic cartoon showing the proposing action of LASSBio-897 as a positive allosteric modulator across an A_2A_ dimer. **(A)** Representative docking of LASSBio-897 showing the interaction region at the orthosteric site (green) and at the allosteric site (red). The sodium ion (yellow) is shown for comparison. **(B)** Schematic cartoon representing the normal action of adenosine at the orthosteric site (green) of A_2A_. **(C)** Schematic cartoon representing the dual orthosteric (green)/allosteric (red) mode of interaction predicted by LASSBio-897 at a dimeric A_2A_, being able to bind in a bitopic manner to one protomer and exert positive cooperativity across the dimer for the binding of adenosine to the other protomer. AD, adenosine; L-897, LASSBio-897.

In order to test the hypothesis of a positive modulator allosteric effect of LASSBio-897, we performed a molecular docking study using the crystal structure of the A_2A_ receptor with PDB code 4EIY (Liu et al., 2012). The docking analysis showed that LASSBio-897 has potential to bind at both orthosteric and sodium ion allosteric sites of the A_2A_ receptor. Since the pre-treatment with amiloride, a negative allosteric modulator, abolished the synergistic effect of LASSBio-897 on adenosine-induced cAMP, we propose that LASSBio-897 modulates positively the A_2A_ receptor activity by binding at the sodium ion allosteric site. A putative molecular mechanism of action that could integrate all our data could be suggested according to what had been proposed by the Christopoulos’s group (Lane et al., 2014) for their data on SB269652 effect on a dimeric D_2_ receptor complex. Accordingly, we are suggesting as a working hypothesis that LASSBio-897 could be a bitopic ligand able to bind on one A_2A_ receptor protomer to modulate allosterically the effect of adenosine to the orthosteric site of a second protomer, considering the plausible existence of homodimers of this receptor in our assays. This hypothesis could explain the displacement of agonist ligands in our binding assays (competition for the orthosteric site) and also the positive allosteric modulation in the cAMP assay, considering that this effect is mainly an increase in efficacy of adenosine (**Figure 12**).

The fact that LASSBio-897 is able to increase the adenosine-induced activation of A_2A_ receptor is indeed interesting, since adenosine accumulates extracellularly in inflamed and fibrotic tissues through rapid conversion of adenine nucleotides released from many cells, including mast cells, nerves and endothelial cells, to adenosine (Linden, 2001; Sitkovsky and Lukashev, 2005). Furthermore, extracellular adenosine stores at inflammatory tissue usually act as an organ protection (Linden, 2001). During inflammation, the defensive action of adenosine occurs through the activation of A_2A_ receptors, which culminates in anti-inflammatory and pro-resolution responses, including inhibition of pro-inflammatory cytokines TNF-α and IL-6 and augmentation of anti-inflammatory cytokine IL-10 (Pinhal-Enfield et al., 2003), besides enhancing alternative macrophage activation (Koroskenyi et al., 2011; Csoka et al., 2012). In addition, although the majority of works described the anti-inflammatory effect of A_2A_ receptor using orthosteric agonists, it has been shown that allosteric ligands able to modulate positively the A_2A_ receptor reduced LPS-induced mouse model of inflammation (Welihinda and Amento, 2014).

Taken together, our findings show that therapeutic treatment with LASSBio-897 reduced granulomatous inflammation and fibrosis into the lungs, associated with improvement of lung function in silicotic mice. LASSBio-897 may represent a promising therapeutic strategy to control silicosis, probably by acting at the A_2A_ receptor through a new mechanism involving synergism with the endogenous agonist.

## Author Contributions

VC: Contributions to design of the work, acquisition and analysis of data, illustration, revision for intellectual content and final approval. TF: Acquisition and analysis of data, illustration, revision for intellectual content and final approval. AdA: Acquisition and analysis of data, illustration and final approval. FN: Acquisition and analysis of data, illustration, critical revision and final approval. RT and CF: Acquisition and analysis of data, illustration, revision for important intellectual content and final approval. CS’A: Acquisition and analysis of data, revision for important intellectual content, supervision and final approval. EB: Revision for important intellectual content, supervision and final approval. PR: Contributions to design of the work, illustration, revision for important intellectual content, supervision and final approval. MM: Design of the work, illustration, drafting of the manuscript, supervision and final approval.

## Conflict of Interest Statement

The authors declare that the research was conducted in the absence of any commercial or financial relationships that could be construed as a potential conflict of interest.
